# Midterm Outcomes of Unicompartmental Knee Arthroplasty for the Treatment of Knee Medial Compartment Osteoarthritis: A Retrospective Study

**DOI:** 10.22038/ABJS.2023.66905.3186

**Published:** 2023

**Authors:** Abolfazl Bagherifard, Mahmoud Jabalameli, Hooman Yahyazadeh, Mohsen Ostovar

**Affiliations:** 1 **Bone and Joint Reconstruction Research Center, Department of Orthopedics, School of Medicine, Iran University of Medical Sciences, Tehran, Iran**; 2 **Department of Orthopedic surgery, Farhikhtegan Hospital, Faculty of Medicine, Tehran Medical Sciences Islamic Azad University, Tehran, Iran**

**Keywords:** Knee osteoarthritic, Medial compartment Unicompartmental knee arthroplasty

## Abstract

**Objectives:**

Many surgeons avoid performing unicompartmental knee arthroplasty (UKA) due to various concerns. Cohort studies showing the satisfactory outcomes of UKA can convince surgeons to use this technique. In this study, we report the mid-term outcomes of UKA in a series of patients with medial compartment knee osteoarthritis.

**Methods:**

Seventeen patients with unicompartmental degenerative joint disease of the knee that underwent UKA and were available for final evaluation were included. The mean age of the patients was 63 ± 5.1 years. The mean follow-up of the patients was 37.2 ± 18.3 months. The outcome measures were the Oxford Knee Score (OKS), Knee Society Score (KSS) for knee score and knee function, Knee injury and Osteoarthritis Outcome Score (KOOS), knee range of motion (ROM), and satisfaction rate on a 5-point Likert scale.

**Results:**

In the last follow-up visit, the mean of OKS and knee score section of the KSS were 44.6 ± 3.2 and 83.8 ± 2.1, respectively. The mean knee function section of the KSS was measured at 98.2 ± 7.2. The mean KOOS score and the mean knee ROM were 84 ± 9.4 and 134.4 ± 7º, respectively. The mean VAS for pain was 8.9 ± 1.1 (range 8-10) before the operation and 1.2 ± 0.8 (range 0-2) at the last follow-up. All the patients were very satisfied (n=14) or satisfied (n=3) with the results. No postoperative complication or reoperation was recorded during the follow-up.

**Conclusion:**

Unicompartmental knee arthroplasty provides satisfactory outcomes and a high survival rate, at least in mid-term follow-up. These findings suggest increased use of UKA in future workups.

## Introduction

Knee replacement is the most commonly used treatment for end-stage knee osteoarthritis (OA) and can be performed as total knee arthroplasty (TKA) and unicompartmental knee arthroplasty (UKA).^1^ Total knee arthroplasty is a highly effective intervention and provides significant improvement in pain, function, and quality of life in knee osteoarthritis (OA) patients.^2^^,^^3^However, UKA has attracted more popularity over the last years because it is less invasive and more closely mimics the normal kinematics of the knee, thereby causing less morbidity and allowing for earlier mobilization and rehabilitation of the patients. ^4^ Despite these potential advantages, preliminary studies have demonstrated a high rate of UKA failure and conversion to TKA.^5^^,^^6^

With the recent advancements in implant design, surgical procedures, and surgical indications, UKA has regained its attraction.^7^^, ^^8^ Even so, many surgeons still avoid this procedure, mainly due to concerns regarding lower prosthesis survivability.^7^ Cohort studies showing the satisfactory outcomes of the UKA are available tools to convince the surgeon to use UKA more widely for patients with partial knee joint involvement. To this aim, we report the mid-term outcomes of UKA in a series of 17 patients with medial compartment knee OA. 

## Materials and Methods

This study was approved by the review board of our institute under the code IR.IUMS.REC.1401.112. Patients provided written informed consent before participation in the study. Between 2016 and 2021, the medical profiles of the patients who underwent UKA for the knee OA treatment were retrospectively reviewed. Inclusion criteria were the age of > 50 years, grade 2-4 medial compartment knee OA according to the Kellgren-Lawrence Classification,^9^ a minimum follow-up of six months, complete medical records, and attendance at the final evaluation session. Patients with inflammatory arthritis, anterior cruciate ligament (ACL) incompetence, degeneration of the patellar joint in the preoperative radiography, flexion contracture of ≥ 15°, knee range of motion of ≤ 90°, angular deformity of > 10° for varus knees and > 5° for valgus knees, and body max index (BMI) ≥ 30 kg/m2 were excluded from the study. Anterior cruciate ligament incompetence was checked clinically and radiologically. The anterior drawer test and the Lachman test were used for the clinical evaluation of ACL deficiency. Anterior tibial subluxation in the plain radiographs and MRI was regarded as the radiologic criteria for ACL incompetence. 

Of 20 patients who were identified as eligible for the study, 17 patients attended the final evaluation session and were included in the final analysis. The study population included three males and 14 females with a mean age of 63 ± 5.1 years (range 54-70). The mean follow-up of the patients was 37.2 ± 18.3 months (range 6-72). The characteristic features of the patients are demonstrated in more detail in [Table 1].

**Table 1:** Demographic characteristics of the patients who underwent unicompartmental knee arthroplasty for the knee osteoarthritis

**Variable**	**Mean ± SD or number (%)**
**Age (year)**	63 ± 5.1
**BMI (kg/m** ^2^ **)**	27.1 ± 3.3
**Sex** **Male ** **Female**	3 (17.6) 14 (82.4)
**Laterality** **Right ** **Left**	8 (47) 9 (53)
**Preoperative VAS for pain**	8.9 ± 1.1
**Follow-up (month)**	37.2 ± 18.3

BMI; Body mass index; VAS: Visual analog scale

 All the surgeries were performed by two senior knee surgeons at a single institution. The type of prosthesis used for UKA was the Zimmer Unicompartmental Knee System (Biomet, Warsaw, IN, USA) mobile bearing. Briefly, a small skin incision was made over the medial side of the patella, starting from the superior border of the patella and ending at the inferior border of the joint line. Then, the joint was exposed through a medial parapatellar arthrotomy, and after osteophyte removal, proximal tibial resection was performed using an extramedullary tibial resection guide. Subsequently, a femoral drill guide and a femoral cut block were used to resect the posterior condyle of the femur. After equalization of the flexion and extension gaps by milling of the distal femoral condyle, the tibial and femoral components were fixed in place with bone cement, while a mobile polyethylene bearing was implemented between them.

 The outcome measures the knee function, anterior knee pain, patients' satisfaction, knee range of motion, postoperative complications, and revision rate. The knee function was evaluated by different questionnaires, including the Oxford Knee Score (OKS), Knee Society Score (KSS), Knee injury and Osteoarthritis Outcome Score (KOOS). When possible, the Persian translation of the questionnaire was used.^10^^,^^11^ The OKS score ranged from 0 to 48. The KSS was presented in two sections (knee and functional scores), both of which were scored from 0 to 100. The KOOS ranged from 0 to 100. In all questionnaires, a higher score was indicative of fewer knee problems. Knee pain was evaluated using the visual analog scale (VAS) for pain on a 0–10 scale, representing a continuum between “no pain” and “extreme pain”, respectively. 

 The patients’ satisfaction was assessed with a 5-point Likert scale for satisfaction that was categorized into very satisfied, satisfied, neutral, dissatisfied, and very dissatisfied. The knee range of motion was measured with a goniometer. All the outcome measures were evaluated by a researcher who was not involved in the patient’s treatment. The postoperative complications were extracted from the patient’s medical records. 

## Results

Based on the findings of the present research, the mean OKS of the patients and the mean knee score section of the KSS were 44.6 ± 3.2 (range 37-48) and 83.8 ± 2.1 (range 80-85), respectively. The mean knee function section of the KSS was measured at 98.2 ± 7.2 (range 70-100). The mean KOOS score of the patients was 84 ± 9.4 (range 69-99). The mean VAS for pain was 8.9±1.1 (range 8-10) before the operation and 1.2±0.8 (range 0-2) at the last follow-up. Moreover, the mean knee range of motion was calculated at 134.4 ±7º (range 120-140).

 Out of 17 patients, 14 (82.3%) patients were very satisfied with the results of the UKA, while the remaining three patients (17.7%) were satisfied according to the 5-point Likert scale. Outcome measures are summarized in [Table 2].

 No postoperative complication was recorded during the follow-up period of the study. Moreover, no patient required revision surgery during the follow-up course.

## Discussion

**In this study, we evaluated the clinical and functional outcomes, satisfaction level, knee pain, postoperative complications, and revision rate following the UKA in patients with medial compartment knee OA. The knee function was acceptable using four different questionnaires. The knee ROM was full or near full in all patients. The anterior knee pain was remarkably** **reduced. **

**All patients were satisfied or very satisfied with the results of UKA. No postoperative complications or revision surgery was recorded in this series.**

**Table 2:** Outcome measures of the patients with unicompartmental knee arthroplasty evaluated in the last follow-up visit

**Variable**	**Mean ± SD or number (%)**
**OKS**	44.6 ± 3.2
**KSS knee score**	83.8 ± 2.1
**KSS knee function**	98.2 ± 7.2
**KOOS**	84 ± 9.4
**VAS for pain**	1.2 ± 0.8
**Knee ROM (** **º** **)**	134.4 ± 7
**Satisfaction** **Very satisfied** **Satisfied** **Neutral** **Dissatisfied** **Very dissatisfied**	14 (82.3) 3 (17.7) - - -

*OKS: Oxford knee score; KSS: Knee society score; IKDC: International Knee Documentation Committee; KOOS: Knee injury and Osteoarthritis Outcome Score; VAS: Visual Analog Scale; ROM: Range of motion.

**The outcomes of UKA have been reported in some earlier studies. In 1980, Insall and Aglietti reported the outcomes of five to seven-year follow-ups of UKA in 22 knees (17 medial and five lateral condyles OA). Although the UKA results were initially favorable, they showed a marked deterioration over time, so only one knee was rated as excellent in the last follow-up, while seven knees were rated as good; four knees were rated as fair, and ten knees were rated as poor. In addition, seven knees were converted to a bicondylar prosthesis.** ^5^ **In 1981, Marmor reported the outcomes of UKA in 60 knees with a minimum follow-up of 10 years. Unicompartmental knee arthroplasty results were excellent in 30 patients, good in eight patients, fair in four patients, and poor in 18 patients. The satisfaction rate and pain reliefs were 70% and 86.6%, respectively. Twenty-one failures were recorded in this series, mainly caused by material or technical problems or improper selection of the patients.** ^12^

**In later years, researchers focused on the improvement of implant design, surgical procedures, and surgical indications.** ^7^^,^^8^ **Parallel to these refinements, the outcomes of UKA also continuously improved. In 2018, Kim et al. reported the long-term outcome of UKA in 80 patients with a mean age of 54.2 years and a mean follow-up period of 12.1 years. The mean KSS knee score and function improved from 52.8 and 56.6 points to 85.4 and 84.7 points, respectively. The mean range of motion improved** **from 130.7° to 132.8°. Postoperative complications were recorded in 20 (16.7%) patients, with mobile-bearing dislocation as the most frequent one (n=9). Ten-year survival (no conversion to TKA) was 92.8%. **^13^

**In 2020, Jansen et al. compared satisfaction rate and functional outcomes of UKA (n=135) with TKA (n=135). The patients were matched for age, sex, BMI, American Society of Anesthesiologists Physical Status classification, and OA grade. At a minimum 1-year follow-up, the UKA group showed significantly less pain, a higher activity level, and a greater satisfaction rate. In addition, UKA patients were able to walk for a longer amount of time without discomfort compared with that in the TKA group. Moreover, the “satisfied or very satisfied” patients were significantly more in the UKA group.** ^14^ **Pandit et al. evaluated the outcomes of 1,000 UKA patients with a mean follow-up of 5.6 years. The mean KSS knee and functional scores were 86.4 and 86.1, respectively. A total of 29 (2.9%) patients required reoperation. Accordingly, the ten-year survival rate was calculated as 96%. The most common cause of failures was arthritis in the lateral compartment, dislocation of the bearing, and unexplained pain.** ^15^ **Newman et al. compared the functional outcomes and survival rate of UKA with TKA in 15-year results of a prospective randomized controlled trial. Bristol knee scores were remarkably better in the UKA group. The 15-year survival rate was 89.8% and 78.7% for UKA and TKA, respectively.** ^16^

**In the present study, UKA provided an acceptable function and satisfaction rate. None of our patients required revision surgery during the follow-up period; therefore, a UKA survival rate of 100% was obtained within a mean follow-up of three years. Such a small rate of failure could be attributed to the meticulous selection of the patients in the present study. Recent improvements in the prosthesis design and procedure could have also attributed to the reduction of UKA failure, thereby justifying wider use of UKA in future workups.**

**The present study was not without limitations. The main limitations of the study were its retrospective design, a small number of patients, and a short duration of follow-up. Therefore, future prospective studies with larger patients and longer follow-up periods are required to confirm the results presented here.**

## Conclusion

**Unicompartmental knee arthroplasty provides acceptable clinical-functional outcomes and satisfaction rates in patients with medial compartment knee OA, at least in mid-term follow-up. With the recent advancement in UKA implant design, procedure, and indications, the rate of UKA failure has significantly reduced. These findings suggest the wider use of UKA for the treatment of unicompartmental knee OA in future workups.**
